# Associations of Changes in Religiosity With Flourishing During the COVID-19 Pandemic: A Study of Faith Communities in the United States

**DOI:** 10.3389/fpsyg.2022.805785

**Published:** 2022-04-05

**Authors:** Christopher Justin Jacobi, Richard G. Cowden, Brandon Vaidyanathan

**Affiliations:** ^1^Nuffield College, Oxford University, Oxford, United Kingdom; ^2^Human Flourishing Program, Harvard University, Cambridge, MA, United States; ^3^Department of Sociology, The Catholic University of America, Washington, DC, United States

**Keywords:** flourishing, religiosity, COVID-19, faith community, well-being

## Abstract

This study explored the extent to which perceived changes in religiosity from before to during the COVID-19 pandemic are associated with flourishing. Participants from a diverse set of faith communities in two United States metropolitan regions (*N* = 1,480) completed an online survey between October and December 2020. The survey included items capturing perceived changes in four dimensions of religiosity (i.e., importance of religion, frequency of prayer, frequency of religious service attendance, and sense of connectedness to one’s faith community) and a multidimensional measure of flourishing. Based on multilevel regressions, results indicated that self-reported decreases in each dimension of religiosity were associated with lower overall flourishing. This pattern of findings was largely similar for the domains of flourishing, with some variation in the strength of associations that emerged. An increase in frequency of religious service attendance was associated with lower overall flourishing and lower scores on selected domains of flourishing (e.g., mental and physical health), indicating possible evidence of religious coping. Faith communities might have to find ways of supporting members during the challenging COVID-19 period to prevent long-term declines in flourishing.

## Introduction

Considerable research finds positive associations between religiosity and various dimensions of wellbeing, including physical health, mental health, life satisfaction, and happiness (Lim and Putnam, 2010; Koenig et al., 2012; VanderWeele, 2017a). The COVID-19 pandemic, however, has necessitated restrictions on religious services and activities. Given the concerns of a global mental health crisis due to the pandemic (Pfefferbaum and North, 2020), it is essential to examine the extent to which the pandemic has brought about changes in religious practices that might impact various dimensions of human flourishing.

Several dimensions of religiosity are relevant to human flourishing. Public religious practice (i.e., religious service attendance) is strongly associated with reduced mortality (Chida et al., 2009), better mental health (Ellison et al., 2009; Li et al., 2016; VanderWeele et al., 2016), better social relationships (Wilcox and Wolfinger, 2016), life satisfaction (Myers, 2008), as well as virtuous and prosocial behavior (Putnam and Campbell, 2012). Private religious practices, such as prayer and meditation, are found to be associated with greater self-esteem (Krause, 2004; You et al., 2019), sustained decreases in depression and anxiety (Boelens et al., 2012), and long-term decreases in pain (Beiranvand et al., 2014). Religious salience, or the importance of religion in one’s life, has also been found to be positively related to mental health (Stroope et al., 2019; Hodge, 2020; Milstein et al., 2020). Finally, research emphasizes the importance of religious social support in shaping various aspects of wellbeing, especially among older adults who feel connected to a community in which they experience spiritual and emotional support (Krause et al., 2001; Krause and Hayward, 2014).

Some studies find negative relationships between the above dimensions of religion and mental health, which may be due to multivalency in different dimensions of religion and the inability to ascertain causal direction in cross-sectional studies (Schieman et al., 2013). Research also suggests that the protective function of religion for wellbeing during adversity may vary based on the nature of the stressor. For example, some studies (e.g., Strawbridge et al., 1997; Bradshaw and Ellison, 2010) have found that religious service attendance may buffer the negative effects of some stressors (e.g., financial hardship) on mental health but not others (e.g., marital conflict). The COVID-19 pandemic is an especially powerful stressor and provides a unique opportunity to assess religion’s relationship with various indicators of wellbeing. Using cross-sectional data collected from congregants of various religious traditions during the COVID-19 pandemic, this study hypothesizes that decreases in (a) the importance of religion in daily life, (b) frequency of prayer, (c) frequency of religious service attendance, and (d) sense of connectedness to one’s faith community will be associated with lower levels of flourishing.

## Methods

Between October and December 2020, an online survey was conducted in 12 diverse urban and suburban congregations in Washington DC, Maryland, Virginia, and Texas. Participating congregations included four Catholic, one Evangelical, three Jewish, two African American Baptist, one Mormon, and one Hindu community. Faith community leaders emailed an online link to the survey to their membership lists. A participation incentive of $1,000 and a report of the main findings were given to each community. This purposive sample of congregations was selected to reflect a diversity of sizes, localities, and traditions, but it is not statistically representative of the denominations or their localities. Despite the limitations of the sample, it is fitting to study the impacts of the COVID-19 pandemic on religiosity and flourishing in a highly religious context.

The four variables for perceived changes in dimensions of religiosity were based on items in response to the question “Overall, how have the following changed (if at all) since the COVID-19 pandemic started?”: (a) “How important your religious faith is to you in your everyday life,” (b) “How often you pray or meditate,” (c) “How often you attend religious services,” and (d) “The sense of connection or closeness you feel toward your local faith community.” Survey respondents were asked to select one of the following response categories for each of the four dimensions of religiosity: “decreased since the pandemic,” “same as before the pandemic,” and “increased since the pandemic.”

Participants completed the Flourishing Index (VanderWeele, 2017b). The measure contains 10 items that are evenly distributed across five domains: (1) life satisfaction and happiness (e.g., “Overall, how satisfied are you with life as a whole these days?”), (2) mental and physical health (e.g., “How would you rate your overall mental health?”), (3) meaning and purpose (e.g., “Overall, to what extent do you feel the things you do in your life are worthwhile?”), (4) character and virtue (e.g., “I always act to promote good in all circumstances, even in difficult and challenging situations”), and (5) close social relationships (e.g., “I am content with my friendships and relationships”). The two statements/questions for each domain are averaged for domain-specific scores that range from 0 to 10, with high scores implying greater wellbeing. An overall flourishing score is also calculated by averaging participants’ responses to all 10 items. In this study, estimated internal consistency reliability for overall flourishing was α = 0.90.

Multivariate analyses control for age (continuous), gender (0: female, 1: male), race (1: White American, 2: Hispanic, 3: Black American, 4: Asian American, 5: other), marital status (0: unmarried, 1: married or in partnership), education (0: less than a college degree, 1: college degree or higher), income (continuous) and political party (1: Republican, 2: Independent, 3: Democrat). We also include a measure of religious service attendance before the COVID-19 pandemic (“Aside from weddings, funerals, and religious holidays, about how often did you normally attend religious services at your faith community before the COVID-19 pandemic started?”). Response options include 1: a few times a year or less, 2: once a month or less, 3: 2–3 times a month, 4: once a week, 5: several times a week. Descriptive statistics of the sample characteristics are presented in Table 1.

**TABLE 1:** Descriptive statistics of the analytic sample (*N* = 1,480).

	Percentage (%)	Mean	SD
*Flourishing Index^a^*		7.5	1.4
Happiness and life satisfaction		7.0	1.8
Mental and physical health		7.4	1.6
Meaning and purpose		7.9	1.7
Character and virtue		7.8	1.4
Close social relationships		7.5	2.0
***Changes in dimensions of religiosity***			
Change in importance of religion*^b^*			
Decreased since the pandemic	2		
Increased since pandemic	24		
Change in frequency of prayer*^b^*			
Decreased since the pandemic	7		
Increased since pandemic	33		
Change in frequency of religious service attendance*^b^*			
Decreased since the pandemic	41		
Increased since pandemic	14		
Change in sense of connectedness to one’s faith community*^b^*			
Decreased since the pandemic	32		
Increased since pandemic	19		
***Control variables***			
Frequency of religious service attendance before the COVID-19 pandemic		3.4	1.4
Gender: men (ref. women)	32		
Race/ethnicity			
White American	71		
African American	10		
Hispanic	9		
Asian American	6		
Other	3		
Age (years)		59.0	13.8
Marital status: married or in partnership (ref. single/divorced/widowed)	78		
Education: college degree or higher (ref. less than a college degree)	83		
Household income (treated as continuous in the regression models)			
Up to $35,000	6		
$35,001–$50,000	6		
$50,001–$100,000	20		
$100,001–$150,000	21		
More than $150,000	47		
Political party			
Republican	23		
Democrat	56		
Independent	21		

*Mental Health in Congregations Study (2020), Washington, D.C., United States. Analytic sample after missing data imputation (N = 1,480). Percentages and means are unweighted.*

*^a^The Flourishing Index and its domains are standardized in the linear multilevel regression models.*

*^b^The percentages for the middle categories of “same as before the pandemic” are not shown but can be deduced when subtracting from 100.*

A series of linear multilevel regression models were estimated, regressing flourishing and its five domains (six outcomes in total) on each of the change in religiosity measures (four predictors in total). A total of 24 models were estimated. The multilevel modeling approach in which individuals (level 1) are nested in congregations (level 2) makes it possible to statistically account for the clustering of individual respondents in the 12 congregations. The number of respondents per congregation ranged from 28 to 148 (*M* = 82). Statistical analyses were conducted in Stata 16.1. Missing data imputations were run *via* machine learning techniques using the *missForest* package in R (see Supplementary Material). The analysis was replicated using complete cases, the results of which were comparable to those produced after imputation (see Supplementary Material). We focus our interpretation on the analysis involving the imputed data.

## Results

Results of the multilevel regression analyses are reported in Table 2. Decreases and increases in religiosity are always compared to the reference group of no changes during the COVID-19 pandemic. Of the four dimensions of religiosity, a decrease in the importance of religion evidenced the strongest negative associations with overall flourishing and each of its domains. Decreases in the importance of religion (β = −1.11, *p* < 0.001), frequency of prayer (β = −0.60, *p* < 0.001), frequency of religious service attendance (β = −0.22, *p* < 0.001), and sense of connectedness to one’s faith community (β = −0.44, *p* < 0.001) were each robustly associated with lower overall flourishing.

**TABLE 2:** Linear multilevel regression models for associations of perceived changes in four dimensions of religiosity with flourishing and each of its domains (*N* = 1,480).

Exposure	Criterion					
	
	Flourishing	Life satisfaction and happiness	Mental and physical health	Meaning and purpose	Character and virtue	Close social relationships
						
	β [95% CI]	β [95% CI]	β [95% CI]	β [95% CI]	β [95% CI]	β [95% CI]
**Change in religious importance**						
Decrease (vs. no change)	−1.11*** [−1.45, −0.77]	−0.81*** [−1.15, −0.47]	−0.90*** [−1.24, −0.56]	−1.08*** [−1.43, −0.74]	−0.79*** [−1.65, −0.64]	−0.80*** [−1.14, −0.45]
Increase (vs. no change)	−0.01 [−0.11, 0.13]	−0.04 [−0.15, 0.08]	0.02 [−0.14, 0.10]	−0.01 [−0.13, 0.11]	0.06 [−0.06, 0.18]	0.03 [−0.09, 0.15]
**Change in frequency of prayer**						
Decrease (vs. no change)	−0.60*** [−0.79, −0.40]	−0.40*** [−0.60, −0.20]	−0.56*** [−0.76, −0.37]	−0.57*** [−0.77, −0.37]	−0.36*** [−0.57, −0.16]	−0.45*** [−0.65, −0.25]
Increase (vs. no change)	−0.08 [−0.19, 0.02]	−0.12* [−0.23, −0.01]	−0.12* [−0.23, −0.01]	−0.04 [−0.15, 0.06]	−0.01 [−0.12, 0.10]	−0.04 [−0.15, 0.07]
**Change in frequency of religious service attendance**						
Decrease (vs. no change)	−0.22*** [−0.32, −0.11]	−0.22*** [−0.33, −0.11]	−0.21*** [−0.32, −0.11]	−0.18*** [−0.29, −0.07]	−0.06 [−0.17, 0.05]	−0.17*** [−0.28, −0.06]
Increase (vs. no change)	−0.20* [−0.35, −0.05]	−0.15 [−0.30, 0.00]	−0.23* [−0.38, −0.07]	−0.13 [−0.29, 0.02]	−0.09 [−0.25, 0.07]	−0.18* [−0.33, −0.02]
**Change in sense of connectedness to one’s faith community**						
Decrease (vs. no change)	−0.44*** [−0.55, −0.32]	−0.36*** [−0.48, −0.25]	−0.32*** [−0.43, −0.21]	−0.36*** [−0.48, −0.25]	−0.13* [−0.25, −0.01]	−0.50*** [−0.61, −0.38]
Increase (vs. no change)	−0.08 [−0.21, 0.05]	−0.07 [−0.20, 0.06]	−0.11 [−0.25, 0.02]	−0.09 [−0.23, 0.04]	0.01 [−0.12, 0.15]	−0.05 [−0.18, 0.08]

*β = standardized regression coefficients, CI = confidence interval. Each criterion variable was regressed on perceived change in each dimension of religiosity in separate models. Linear multilevel (ordinary least) squares regressions were used to estimate associations of perceived changes in each dimension of religiosity with flourishing and each of its domains. All models adjusted for gender, age, marital status, education, household income, political party identification, and frequency of religious service attendance before the COVID-19 pandemic. Based on missing data imputations, all models have a constant sample size of 1,480 (Mental Health in Congregations Study, 2020, Washington, D.C., United States).*

*p < 0.05 before but not after Bonferroni correction, ***p < 0.05 after Bonferroni correction (the p-value cut-off for Bonferroni correction was 0.05/24 = 0.002 for each outcome).

The strengths of the associations of changes in religiosity with the five domains of flourishing generally followed a similar pattern as the associations for overall flourishing. Decreases in both importance of religion (βs = −1.08 to −0.79, *p*s ≤ 0.001) and frequency of prayer (βs = −0.57 to −0.36, *p*s ≤ 0.01) were robustly associated with lower flourishing on all five domains. Robust associations were also found for both a decrease in the frequency of religious service attendance (βs = –0.22 to –0.17, *p*s ≤ 0.01) and for a decrease in sense of connectedness to one’s faith community (βs = –0.50 to –0.32, *p*s ≤ 0.001) with life satisfaction and happiness, mental and physical health, meaning and purpose, and close social relationships. A decrease in sense of connectedness to one’s faith community was modestly associated with the character and virtue domain (β = −0.13, *p* = 0.03), but there was little evidence of an association between a decrease in frequency of religious service attendance and the character and virtue domain (β = −0.06, *p* = 0.26).

There was also modest evidence indicating that an increase in frequency of religious service attendance was associated with lower overall flourishing (β = −0.20, *p* = 0.01). There was little evidence to suggest that increases in religious importance (β = −0.01, *p* = 0.89), frequency of prayer (β = −0.08, *p* = 0.13), and sense of connectedness to one’s faith community (β = −0.08, *p* = 0.23) were associated with overall flourishing.

For the domains of flourishing, modest associations were found between an increase in frequency of religious service attendance and lower mental and physical health and close social relationships (βs = −0.23 to −0.18, *p*s ≤ 0.03). Similarly, an increase in prayer was modestly associated with lower scores on the domains of life satisfaction and happiness and mental and physical health (βs = −0.12, *p*s ≤ 0.04). None of the other associations between increases in the dimensions of religiosity and the flourishing domains reached statistical significance (βs = −0.15 to.06, *p*s > 0.053).

## Discussion

In support of this study’s hypotheses, self-reported declines in religious importance, frequency of prayer, religious service attendance, and closeness to one’s faith community from before to during the COVID-19 pandemic were each associated with lower levels of flourishing. A similar pattern of findings was identified for the domains of flourishing. The largest associations emerged for religious importance, whereas the smallest were found for in-person religious service attendance. Taken together, these findings align with the growing body of literature on the implications of the COVID-19 pandemic for religious/spiritual life (e.g., Cowden et al., 2021b; Davis et al., 2021) and empirical evidence that suggests religious/spiritual life plays an important role in contributing to the wellbeing of many people (e.g., Chen et al., 2020).

The consistently negative associations between the dimensions of religiosity and the domain of close social relationships speak to the impact that the COVID-19 pandemic has had on religious communities (VanderWeele, 2020), and point to the challenges of maintaining cohesive religious communities during a public health crisis. Despite widespread online religious services and opportunities to participate in virtual religious activities organized by faith-based communities (Counted et al., 2020), our findings suggest that parishioners may have found it challenging to sustain their flourishing if their sense of connectedness with other faith community members was disrupted during the COVID-19 pandemic.

Contrary to our predictions, we found modest evidence suggesting that an increase in in-person religious service attendance was associated with lower flourishing (and both the domains of mental and physical health and close social relationships). Similarly, an increase in prayer was modestly associated with both lower life satisfaction and happiness and mental and physical health. These findings could be explained by the *stress mobilization* hypothesis (Pargament, 1997), which refers to the notion that stressors can precipitate religious/spiritual coping responses (e.g., seeking comfort from God through prayer). Those who reported an increase in religiosity may have been seeking religious/spiritual resources that could provide them with some relief from the distress they were experiencing during the early part of the COVID-19 pandemic (Cowden et al., 2021a; Counted et al., 2022).

There are several limitations that should be considered when interpreting the findings of this study. First, our use of cross-sectional data means that we cannot rule out the possibility of reverse causation. Although we retrospectively assessed perceived changes in dimensions of religiosity since the beginning of the COVID-19 pandemic, it is possible that participants’ religiosity could have been influenced by their current levels of flourishing. Longitudinal studies are needed to acquire more robust evidence linking religiosity with flourishing within the context of the COVID-19 pandemic. Second, change in religiosity was assessed using a crude set of items that may not have sufficiently captured changes in religious participation, behavior, or experience. Third, participants were conveniently sampled members of selected religious congregations in the United States. This study’s results may not generalize to the broader population of individuals who belong to religious communities.

## Conclusion

This study has demonstrated that decreases in dimensions of religiosity during the COVID-19 pandemic are associated with lower flourishing, with particularly strong associations found for perceived declines in the importance of religion and frequency of prayer. Congregants who reported an increase in religiosity might benefit from spiritual or mental health support if they turned to religion because of stressors experienced during the public health crisis. In the wake of the COVID-19 pandemic, religious organizations will need to find ways of supporting the religious/spiritual lives of parishioners which would involve both personal aspects (e.g., maintaining meaningful prayer practices) and social aspects (e.g., maintaining connections among members). Future research could build on the findings of this study by examining the ways in which pandemic-related adversity might have led to increases in certain dimensions of religiosity.

## Data Availability Statement

The raw data supporting the conclusions of this article will be made available by the authors, without undue reservation.

## Ethics Statement

The studies involving human participants were reviewed and approved by the Institutional Review Board of the Catholic University of America (protocol number 18-082). The patients/participants provided their written informed consent to participate in this study.

## Author Contributions

CJ contributed to the conceptualization of the manuscript, data management and statistical analyses, as well as writing. RC made contributions with regards to conceptualizing the manuscript, providing analytic support, writing, and revisions. BV contributed funding acquisition, data collection, and writing. All authors contributed to the article and approved the submitted version.

## Conflict of Interest

The authors declare that the research was conducted in the absence of any commercial or financial relationships that could be construed as a potential conflict of interest.

## Publisher’s Note

All claims expressed in this article are solely those of the authors and do not necessarily represent those of their affiliated organizations, or those of the publisher, the editors and the reviewers. Any product that may be evaluated in this article, or claim that may be made by its manufacturer, is not guaranteed or endorsed by the publisher.

## References

[B1] Beiranvand S. Noparast M. Eslamizade N. Saeedikia S. (2014). The effects of religion and spirituality on postoperative pain, hemodynamic functioning and anxiety after cesarean section. *Acta Med. Iranica* 52 909–915. 25530054

[B2] Boelens P. A. Reeves R. R. Replogle W. H. Koenig H. G. (2012). The effect of prayer on depression and anxiety: maintenance of positive influence one year after prayer intervention. *Int. J. Psychiatry Med.* 43 85–98. 10.2190/PM.43.1.f 22641932

[B3] Bradshaw M. Ellison C. G. (2010). Financial hardship and psychological distress: exploring the buffering effects of religion. *Soc. Sci. Med.* 71 196–204. 10.1016/j.socscimed.2010.03.015 20556889 PMC3770858

[B4] Chen Y. Kim E. S. VanderWeele T. J. (2020). Religious-service attendance and subsequent health and well-being throughout adulthood: evidence from three prospective cohorts. *Int. J. Epidemiol.* 49 2030–2040. 10.1093/ije/dyaa120 32793951 PMC7825951

[B5] Chida Y. Steptoe A. Powell L. H. (2009). Religiosity/spirituality and mortality: a systematic quantitative review. *Psychotherapy Psychosomat.* 78 81–90. 10.1159/000190791 19142047

[B6] Counted V. Neff M. A. Captari L. E. Cowden R. G. (2020). Transcending place attachment disruptions during a public health crisis: spiritual struggles, resilience, and transformation. *J. Psychol. Christianity* 39 276–287.

[B7] Counted V. Pargament K. I. Bechara A. O. Joynt S. Cowden R. G. (2022). Hope and well-being in vulnerable contexts during the COVID-19 pandemic: does religious coping matter? *J. Positive Psychol.* 17 70–81. 10.1080/17439760.2020.1832247

[B8] Cowden R. G. Counted V. Ramkissoon H. (2021a). “Adapting to place attachment disruption during a pandemic: from resource loss to resilience,” in *Place and Post-Pandemic Flourishing* (Cham: Springer), 71–80. 10.1007/978-3-030-82580-5_6

[B9] Cowden R. G. Rueger S. Y. Davis E. B. Counted V. Kent B. V. Chen Y. (2021b). Resource loss, positive religious coping, and suffering during the COVID-19 pandemic: a prospective cohort study of US adults with chronic illness. *Ment. Health Relig. Cult.* Online ahead of print. 10.1080/13674676.2021.1948000

[B10] Davis E. B. McElroy-Heltzel S. E. Lemke A. W. Cowden R. G. VanderWeele T. J. Worthington E. L. (2021). Psychological and spiritual outcomes during the COVID-19 pandemic: a prospective longitudinal study of adults with chronic disease. *Health Psychol.* 40 347–356. 10.1037/hea0001079 34323537

[B11] Ellison C. G. Burdette A. M. Hill T. D. (2009). Blessed assurance: religion, anxiety, and tranquility among US adults. *Soc. Sci. Res.* 38 656–667. 10.1016/j.ssresearch.2009.02.002 19856703

[B12] Hodge D. R. (2020). Religious congregations: an important vehicle for alleviating human suffering and fostering wellness. *J. Religion Spirituality Soc. Work: Soc. Thought* 39:119137.

[B13] Koenig H. G. King D. E. Carson V. B. (2012). *Handbook of Religion and Health*, 2nd Edn. Oxford: Oxford University Press.

[B14] Krause N. (2004). Assessing the relationships among prayer expectancies, race, and self-esteem in late life. *J. Sci. Study Religion* 43 395–408. 10.1093/geronb/58.3.s160 12730317

[B15] Krause N. Hayward R. (2014). Church-based social support, religious commitment, and health among older Mexican Americans. *J. Soc. Personal Relationships* 31 352–365. 10.1177/0265407513494952

[B16] Krause N. Ellison C. G. Shaw B. A. Marcum J. P. Boardman J. D. (2001). Church-based social support and religious coping. *J. Sci. Study Religion* 40 637–656. 10.1111/0021-8294.00082

[B17] Li S. Okereke O. I. Chang S. C. Kawachi I. VanderWeele T. J. (2016). Religious service attendance and lower depression among women: a prospective cohort study. *Ann. Behav. Med.* 50 876–884. 10.1007/s12160-016-9813-9 27393076 PMC5127763

[B18] Lim C. Putnam R. D. (2010). Religion, social networks, and life satisfaction. *Am. Sociol. Rev.* 75 914–933. 10.1177/0003122410386686

[B19] Milstein G. Palitsky R. Cuevas A. (2020). The religion variable in community health promotion and illness prevention. *J. Prevention Intervention Community* 48 1–6. 10.1080/10852352.2019.1617519 31402789

[B20] Myers D. G. (2008). “Religion and human flourishing,” in *The Science of Subjective Well-being*, eds Eid M. Larsen R. J. (New York, NY: Guilford), 323–346. 10.1016/j.ajp.2012.09.010

[B21] Pargament K. I. (1997). *The Psychology of Religion and Coping: Theory, Research, Practice.* New York, NY: Guilford Press.

[B22] Pfefferbaum B. North C. S. (2020). Mental health and the Covid-19 pandemic. *New England J. Med.* 383 510–512. 32283003 10.1056/NEJMp2008017

[B23] Putnam R. D. Campbell D. E. (2012). *American Grace.* New York, NY: Simon & Schuster.

[B24] Schieman S. Bierman A. Ellison C. G. (2013). “Religion and mental health,” in *Handbook of the Sociology of Mental Health*, eds Aneshensel C. S. Phelan J. C. Bierman A. (Dordrecht: Springer), 10.1007/978-94-007-4276-5_22

[B25] Strawbridge W. J. Cohen R. D. Shema S. J. Kaplan G. A. (1997). Frequent attendance at religious services and mortality over 28 years. *Am. J. Public Health* 87, 957–961. 10.2105/ajph.87.6.957 9224176 PMC1380930

[B26] Stroope S. Kent B. V. Zhang Y. Spiegelman D. Kandula N. R. Schachter A. B. (2019). Mental health and self-rated health among US South Asians: the role of religious group involvement. *Ethnicity Health.* Online ahead of print. 10.1080/13557858.2019.1661358 31466458 PMC7048668

[B27] VanderWeele T. J. (2017a). Religious communities and human flourishing. *Curr. Direct. Psychol. Sci.* 26 476–481. 10.1177/0963721417721526 29170606 PMC5665144

[B28] VanderWeele T. J. (2017b). On the promotion of human flourishing. *Proc. Natl. Acad. Sci. U S A.* 114 8148–8156. 10.1073/pnas.1702996114 28705870 PMC5547610

[B29] VanderWeele T. J. (2020). Love of neighbor during a pandemic: navigating the competing goods of religious gatherings and physical health. *J. Religion Health* 59 2196–2202. 10.1007/s10943-020-01031-6 32405925 PMC7218704

[B30] VanderWeele T. J. Jackson J. W. Li S. (2016). Causal inference and time-varying exposures: a case study of religion and mental health. *Soc. Psychiatry Psychiatric Epidemiol.* 51 1457–1466. 10.1007/s00127-016-1281-9 27631394

[B31] Wilcox W. B. Wolfinger N. H. (2016). *Soul Mates: Religion, Sex, Love, and Marriage Among African Americans and Latinos.* New York, NY: Oxford University Press. 10.1016/j.jnma.2016.08.007 27979010

[B32] You S. Yoo J. E. Koh Y. (2019). Religious practices and mental health outcomes among Korean adults. *Personal. Individual Differ.* 142 7–12. 10.1016/J.PAID.2019.01.026

