# Supplementary material for: Associations of Changes in Religiosity With Flourishing During the COVID-19 Pandemic: A Study of Faith Communities in the United States
Source: Front Psychol. 2022 Apr 5;13:805785. doi: 10.3389/fpsyg.2022.805785 (PMC9016175; doi:10.3389/fpsyg.2022.805785)
Supplement: Supplementary file 1 [file Table_1.DOCX]

**Associations of Changes in Religiosity and Flourishing During the COVID-19 Pandemic: A Study of Faith Communities in the U.S.**

**[DOI: 10.3389/fpsyg.2022.805785]**

**Supplementary Material 1: Graph of changes in the four dimensions of religiosity from before to during the COVID-19 pandemic**

**
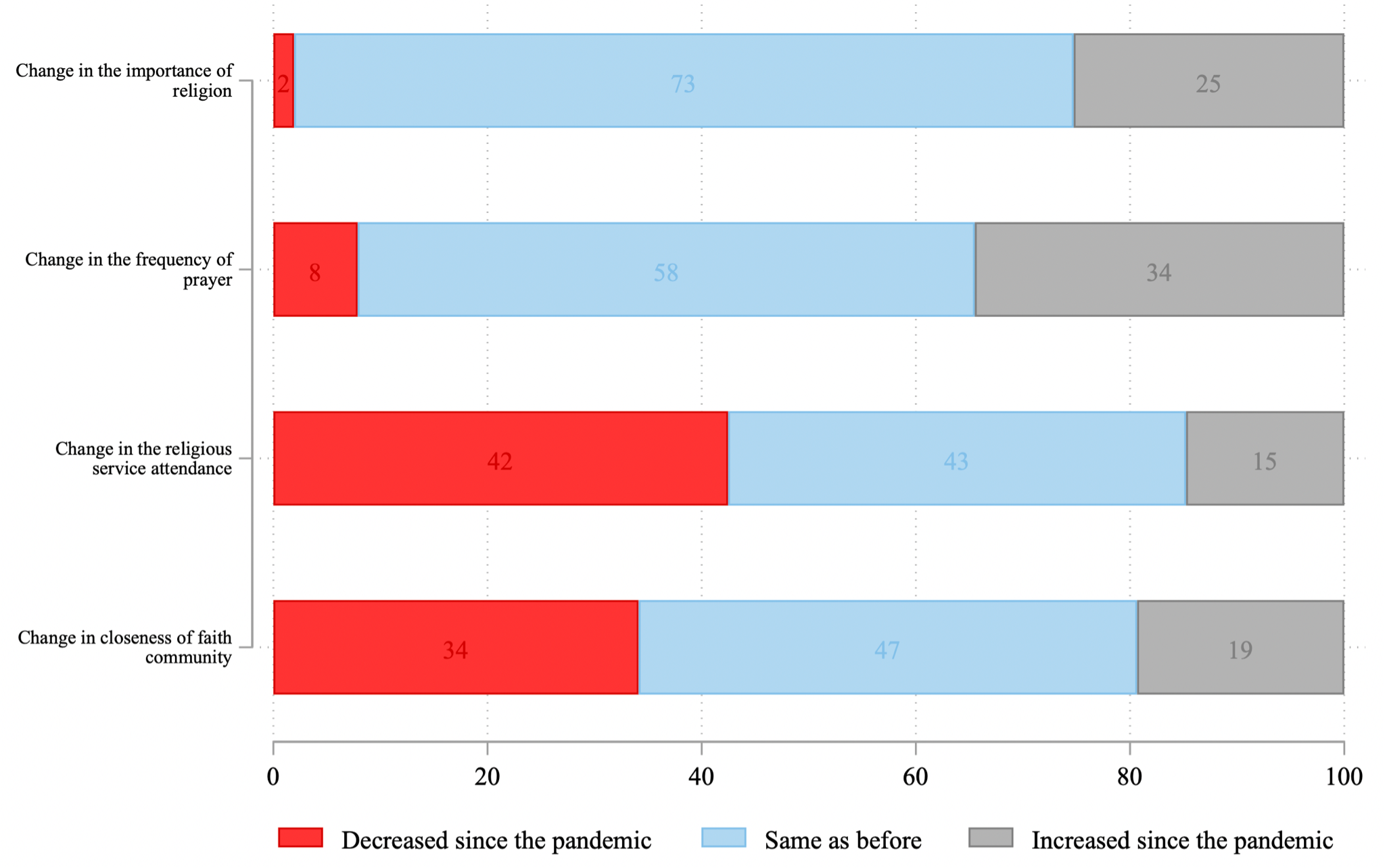
**

Note: Mental Health in Congregations Study (2020). N=1,480.
