# Supplementary material for: Associations of Changes in Religiosity With Flourishing During the COVID-19 Pandemic: A Study of Faith Communities in the United States
Source: Front Psychol. 2022 Apr 5;13:805785. doi: 10.3389/fpsyg.2022.805785 (PMC9016175; doi:10.3389/fpsyg.2022.805785)
Supplement: Supplementary file 2 [file Table_2.DOCX]

**Associations of Changes in Religiosity and Flourishing During the COVID-19 Pandemic: A Study of Faith Communities in the U.S.**

**[DOI: 10.3389/fpsyg.2022.805785]**

**Supplementary Material 2: Details of the Flourishing Index**

Harvard Flourishing Index
(0-10 scales)

**Domain 1: Happiness and Life Satisfaction.**

- 1. Overall, how satisfied are you with life as a whole these days? *(0=Not Satisfied at All, 10=Completely Satisfied)*
  2. In general, how happy or unhappy do you usually feel? *(0=Extremely Unhappy, 10=Extremely Happy)*

**Domain 2: Mental and Physical Health.**

- 1. In general, how would you rate your physical health? *(0=Poor, 10=Excellent)*
  2. How would you rate your overall mental health? *(0=Poor, 10=Excellent)*

**Domain 3: Meaning and Purpose.**

- 1. Overall, to what extent do you feel the things you do in your life are worthwhile? *(0=Not at All Worthwhile, 10=Completely Worthwhile)*
  2. I understand my purpose in life. *(0=Strongly Disagree, 10=Strongly Agree)*

**Domain 4: Character and Virtue.**

- 1. I always act to promote good in all circumstances, even in difficult and challenging situations. *(0=Not True of Me, 10=Completely True of Me)*
  2. I am always able to give up some happiness now for greater happiness later. *(0=Not True of Me, 10=Completely True of Me)*

**Domain 5: Close Social Relationships**

- 1. I am content with my friendships and relationships. *(0=Strongly Disagree, 10=Strongly Agree)*
  2. My relationships are as satisfying as I would want them to be. *(0=Strongly Disagree, 10=Strongly Agree)*

*A sixth domain of “Financial and Material Stability” that is sometimes part of the flourishing index was not used in this study.
