# Supplementary material for: Associations of Changes in Religiosity With Flourishing During the COVID-19 Pandemic: A Study of Faith Communities in the United States
Source: Front Psychol. 2022 Apr 5;13:805785. doi: 10.3389/fpsyg.2022.805785 (PMC9016175; doi:10.3389/fpsyg.2022.805785)
Supplement: Supplementary file 3 [file Table_3.DOCX]

**Associations of Changes in Religiosity and Flourishing During the COVID-19 Pandemic: A Study of Faith Communities in the U.S.**

**[DOI: 10.3389/fpsyg.2022.805785]**

**Supplementary Material 3: Technical note on the missing data imputations**

The raw dataset contained 1,609 cases. The focal outcome variable of flourishing as well as the predictor variables of changes in dimensions of religiosity had few missing data, but a complete case analysis including the socio-demographic control variables would have resulted in a 39% reduction in the sample size.

Item-level missing data imputations were only conducted if respondents had at least one non-missing response on one of the four changes in religiosity variables, at least one non-missing response on one of the ten flourishing indicators, at least one non-missing response on one of the control variables, and if congregation-membership (i.e., the level 2 information in the multilevel models) was not missing. These conditions resulted in a final analytical sample of N=1,480.

The missing data imputations was based on an iterative random forest procedure with 1000 trees. The out of bag error for the categorical variables, i.e., the proportional falsely classified, is 0.43 and the normalized root mean square error for continuous variables is 0.89.
