# Supplementary material for: Associations of Changes in Religiosity With Flourishing During the COVID-19 Pandemic: A Study of Faith Communities in the United States
Source: Front Psychol. 2022 Apr 5;13:805785. doi: 10.3389/fpsyg.2022.805785 (PMC9016175; doi:10.3389/fpsyg.2022.805785)
Supplement: Supplementary file 4 [file Table_4.DOCX]

**Associations of Changes in Religiosity and Flourishing During the COVID-19 Pandemic: A Study of Faith Communities in the U.S.**

**[DOI: 10.3389/fpsyg.2022.805785]**

**Supplementary Material 4**

*Complete-case linear multilevel regression models for associations of perceived changes in four dimensions of religiosity with flourishing and each of its domains (N=987)*

|  | | | | | | | | | | | | |
| --- | --- | --- | --- | --- | --- | --- | --- | --- | --- | --- | --- | --- |
| Exposure | Criterion | | | | | | | | | | | |
|  | Flourishing |  | Life satisfaction and happiness |  | Mental and physical health |  | Meaning and purpose |  | Character and virtue |  | Close social relationships |  |
|  | β [95% CI] |  | β [95% CI] |  | β [95% CI] |  | β [95% CI] |  | β [95% CI] |  | β [95% CI] |  |
| Change in religious importance |  |  |  |  |  |  |  |  |  |  |  |  |
| Decrease (vs. no change) | -0.95***  [-1.26,-0.63] |  | -1.08*** [-1.50,-0.67] |  | -0.81***  [-1.20,-0.42] |  | -1.20***  [-1.61,-0.79] |  | -0.79***  [-1.19,-0.39] |  | -0.83*** [-1.27,-0.39] |  |
| Increase (vs. no change) | -0.03  [-0.08,0.13] |  | -0.01  [-0.14,0.12] |  | 0.02  [-0.10,0.15] |  | -0.01  [-0.13,0.14] |  | 0.07  [-0.06,0.20] |  | 0.05  [-0.09,0.19] |  |
| Change in frequency of prayer |  |  |  |  |  |  |  |  |  |  |  |  |
| Decrease (vs. no change) | -0.48***  [-0.65,-0.30] |  | -0.45***  [-0.67,-0.22] |  | -0.39***  [-0.60,-0.18] |  | -0.61***  [-0.83,-0.39] |  | -0.49***  [-0.70,-0.27] |  | -0.46*** [-0.69,-0.23] |  |
| Increase (vs. no change) | -0.08  [-0.17,0.01] |  | -0.17*  [-0.30,-0.05] |  | -0.11  [-0.23,0.00] |  | -0.04  [-0.16,0.08] |  | -0.02  [-0.14,0.10] |  | -0.05  [-0.18,0.08] |  |
| Change in frequency of religious service attendance |  |  |  |  |  |  |  |  |  |  |  |  |
| Decrease (vs. no change) | -0.09  [-0.18,0.01] |  | -0.13* [-0.25,-0.01] |  | -0.07  [-0.18,0.05] |  | -0.10  [-0.22,0.02] |  | -0.07  [-0.19,0.05] |  | -0.11  [-0.23,0.02] |  |
| Increase (vs. no change) | -0.13*  [-0.27,-0.00] |  | -0.16  [-0.33,0.02] |  | -0.18*  [-0.34,-0.02] |  | -0.09  [-0.27,0.08] |  | -0.08  [-0.24,0.09] |  | -0.17  [-0.36,0.01] |  |
| Change in sense of connectedness to one’s faith community |  |  |  |  |  |  |  |  |  |  |  |  |
| Decrease (vs. no change) | -0.28***  [-0.38,-0.18] |  | -0.31***  [-0.44,-0.18] |  | -0.25***  [-0.38,-0.13] |  | -0.27***  [-0.40,-0.14] |  | -0.10  [-0.23,0.02] |  | -0.48*** [-0.61,-0.35] |  |
| Increase (vs. no change) | -0.07  [-0.19,0.04] |  | -0.13  [-0.28,0.03] |  | -0.12  [-0.26,0.02] |  | -0.09  [-0.24,0.06] |  | 0.01  [-0.14,0.15] |  | -0.05  [-0.20,0.11] |  |
| *Note*. β = standardized regression coefficients, CI = confidence interval. Each criterion variable was regressed on perceived change in each aspect of religiosity in separate models. Linear multilevel (ordinary least) squares regressions were used to estimate associations of perceived changes in each dimension of religiosity with flourishing and each of its domains. All models adjusted for gender, age, marital status, education, household income, political party identification, and frequency of religious service attendance before the COVID-19 pandemic. Based on listwise missing data deletion, all models have a constant sample size of 987 (Mental Health in Congregations Study 2020). **p* < .05 before but not after Bonferroni correction, ****p* < .05 after Bonferroni correction (the *p*-value cutoff for Bonferroni correction was .05/24 = .002 for each outcome). | | | | | | | | | | | | |
